# Development of a Flexible Dielectric Sensor for Flow Monitoring of the Liquid Resin Infusion Process

**DOI:** 10.3390/s19235292

**Published:** 2019-12-01

**Authors:** Athanasios Pouchias, Paul R. Cunningham, Jasmin Stein, Mihalis Kazilas

**Affiliations:** 1 Department of Aeronautical and Automotive Engineering, Loughborough University, Loughborough LE11 3TU, UK; p.cunningham@lboro.ac.uk; 2 National Structural Integrity Research Centre (NSIRC), Cambridge CB21 6AL, UK; 3 TWI Ltd., Cambridge, CB21 6AL, UK; jasmin.stein@twi.co.uk (J.S.); mihalis.kazilas@twi.co.uk (M.K.); 4 Brunel University London, Kingston Lane, London, Uxbridge UB8 3PH, UK

**Keywords:** dielectric sensors, flexible materials, composite materials, resin flow, partial differential equations, electrostatics

## Abstract

The analysis and design of a novel flexible dielectric sensor, which can be integrated into a composite materials manufacturing process to measure the resin frontal flow, is presented in this paper. The proposed sensor consists of two parallel line electrodes and a ground plane covered by a dielectric material. The analytical description and the electrostatic modelling were considered for the design of the sensor and to enhance the understanding of the response of the sensor to the resin impregnation of a carbon fabric during the infusion phase. The optimization of the sensor’s response and the increase of its sensitivity with regards to the geometric characteristics and the materials used were the main objectives of this study. An experimental set-up for the vacuum infusion process which includes the proposed sensor was used to measure the capacitance and validate the derived resin flow against visual measurements. The results indicate that the sensor can provide information on the resin frontal flow within 2% accuracy against visual measurements, which make this technology promising for monitoring the liquid resin infusion processes.

## 1. Introduction

This paper describes the development of a flexible dielectric sensor that is to be embedded on a pilot Resin Transfer Moulding (RTM) tool for monitoring the resin flow. The main steps of a RTM process for the manufacture of a simple composite part are the fabric pre-form preparation, the draping of the pre-form in the mould tool, the resin injection, and finally the curing phase [1]. The techniques that are used for the manufacturing of composite materials and the variability in process parameters, such as the resin viscosity, pre-form permeability and process conditions, can introduce defects during the manufacturing [2]. Defects such as race tracking and void formation are related to the resin flow. It is for this reason that a reliable method, which includes a robust sensor, is needed to monitor the resin flow in real time and provide a means of identifying any potential faults in the completed composite part. Numerical simulations can also be a useful process design tool providing accurate data is available to describe the problem, in this case permeability of the fabric and viscosity of the resin. However, the key parameters that are used by these models is lacking or is very challenging to obtain [3]. This is particularly true for multi-axis woven fabrics and for that reason, process monitoring is suggested to enhance the understanding of the resin flow through the fabric, within the volume of the mould tool and the final cure.

The highly complex and variable nature of the materials, the process conditions and the geometries add challenges when attempting to monitor and predict the behaviour of the process. Many monitoring technologies have been examined for resin frontal flow and cure progress with spot and global sensors. Spot sensors are used as indicators of the resin arrival. Thermocouples [4], pressure transducers [5,6,7], and optical fibre sensors [8], were implemented to monitor resin flow. These spot sensors can be used for carbon fibre manufacturing, as they are not affected by its conductivity. However, their industrial applicability is constrained due to the complex analysis of the materials properties that they measure. Another point sensor based on resistivity measurements was presented by Danisman et al. [9]. The information provided is limited to the point location which, therefore, requires careful placement of the sensor to critical areas. In complex shape mould geometries this can increase tooling complexity and cost. Global sensors can be linear sensors or organised in a grid structure. Linear sensors, such as the direct current [10,11], dielectric [12] and electric time-domain reflectometry [13] flow sensors were used with glass fabric pre-forms. Resin flow monitoring in the case of carbon fibre materials is the main challenge and will be addressed in this paper. The use of parallel plates as electrodes, as presented by Yenilmez et al. [14], and the sensors arrays [15,16] cannot be applied in the case of carbon fibre materials since the conductivity of the carbon fibres interrupts the electric field and makes flow sensing extremely challenging. Therefore the out-of-plane flow and the reconstruction of a three-dimensional flow with the use of technologies like that presented by Matsuzaki et al. in [16] are not applicable in this study.

Dielectric measurement and analysis has a higher sensitivity compared to other methods with regards to the monitoring of the composite manufacturing process, as it measures the materials complex dielectric permittivity [17]. This measurement is correlated to the resin viscosity, frontal flow and degree of cure [18,19]. The inexpensive dielectric sensors and cost-effective measuring equipment that are used in the dielectric analysis, are advantages to be considered for its industrial applicability. Recently, microscale dielectric coplanar sensors are utilised for the cure monitoring of glass fibre [17,20] and carbon fibre [21] pre-forms. Twisted insulated wires were embedded in carbon fibre pre-forms and were used as a linear dielectric sensor for flow monitoring [22]. In this context, the dielectric analysis appears to be the most promising and popular method for monitoring the resin state in the composite materials manufacturing processes [23,24].

In this work, a linear dielectric sensor that is placed at the outer surface of the fabric was selected to monitor the resin infusion phase of liquid resin composite manufacturing. The main contribution to the changes in the sensor response occurs when the resin covers the surface of the sensor. Then, the response is correlated with the location of the resin providing an indication of the resin frontal flow. The proposed dielectric sensor consists of three copper electrodes covered with a polyimide (Kapton) tape. Two parallel co-planar rectangular shaped electrodes were used for the sensing and a ground plane was used to reduce electromagnetic coupling with the mould [14]. The geometry is similar to that developed by Skordos [12] which was used for resin flow monitoring in glass fabric pre-forms. However, the current work includes the addition of a ground-backplane electrode. This was used as a shielding electrode to minimize the effect of parasitic capacitances. The interference with conductive elements, such as metallic moulds or other electric fields, is reduced as the electric field measures the changes in the material under test (MUT) [25,26,27].

Capacitance is not adequate enough to provide information on the sensor’s robustness to the process. The sensitivity and penetration depth of the sensor are the main characteristics to be considered for sensor optimization [28]. The geometry of the sensor affects both sensitivity and penetration depth. However, in the case of sensing the resin flow during the manufacture of carbon fibre composites, the penetration depth is limited to the resin-rich layer. The sensitivity of the sensor is the main characteristic for increasing the response, therefore it was used as the optimization objective. The geometry optimization was performed with the investigation of the sensor’s geometric characteristics, where the sensor geometry and materials were modelled through an electrostatic analysis using the Partial Differential Equations (PDE) toolbox in Matlab [29]. It was noted that the thickness of the dielectric materials is important for sensitivity [30]. The separation of conductive pre-forms from dielectric sensors with the use of glass cloths was studied for cure monitoring [31]. The use of multi-layer pre-forms to predict permeability and filling time in RTM was presented by Seong et al. [32]. In this study, the addition of a permeable glass fabric material between the sensor and the carbon fabric during the flow monitoring was examined. An experimental set-up was used to obtain the sensor’s capacitance during the resin infusion process and validate the measured changes with the actual flow. The acquired capacitance values were compared with the calculated values through the electrostatic modelling. These values were used to estimate resin frontal flow position which was compared with visual data captured with a camera.

## 2. Design Methodology

### 2.1. Dielectric Sensor Analysis and Sensitivity

**Figure 1:**
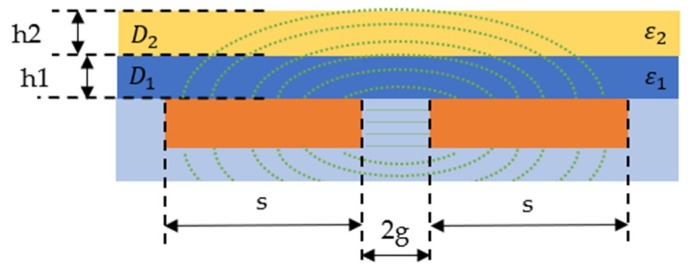
A 2D model of two coplanar electrodes including geometric and material parameters and a representation of the electric field distribution.

Coplanar rectangular electrodes were selected for the dielectric sensor geometry. The movement of the resin during the impregnation phase on the top surface is an indication of the flow progress, and a coplanar capacitive sensor can be analysed with two neighbouring electrodes on the same plane on the top a dielectric layer. The electrodes were covered with a dielectric layer, a polyimide (Kapton) tape to avoid short-circuiting of the electrodes in the case of using conductive carbon fibres. A simplified 2D model with two dielectric materials and the electric field lines are illustrated in Figure 1. The Kapton tape (D1) is the first dielectric material which is in contact with the electrodes. The second dielectric material (D2) can simulate the presence of resin on the top of the sensor. In the case of the conductive carbon fibres which shield the electric field, the second dielectric layer is limited to the resin-rich layer, as described previously. All the parameters of the study and the characteristics which are shown in Figure 1 are described in Table 1.

The capacitance of a coplanar sensor with two dielectric materials has been considered previously by many authors [33,34,35,36,37,38]. A good approximation of the capacitance calculation for the coplanar strip electrodes model is the conformal mapping technique (CMT) [28,30]. In the CMT analysis, the electrodes are considered to have zero thickness and infinite conductivity and the end effects are excluded from the analysis, as the strip length (l) is considered to be much larger than the size of the electrodes (l≫s) [34]. The capacitance between coplanar electrodes with two dielectric layers uses the elliptic integral functions and is given by Equation (1).

(1) $$C=\varepsilon_{0}\cdot \varepsilon_{eff}\cdot \frac{K\left(k_{0}^{\prime }\right)}{K\left(k_{0}\right)}$$

where $K\left(k_{0}\right)$ is the complete elliptic integral of the first kind, $k_{0}$ is the modulus of the elliptic integral function and $\varepsilon_{eff}$ the material’s effective permittivity.

**Figure 2:**
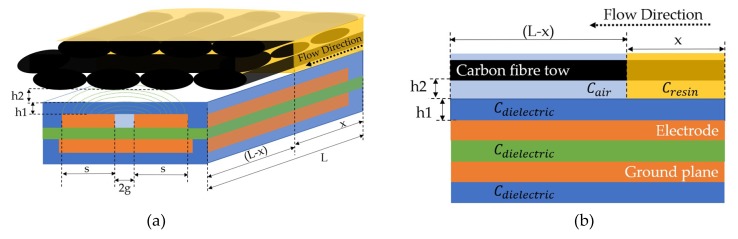
Geometry of the sensor and the parameters that were used in the analytical model: (**a**) Perspective view and dimensions of the sensor geometry; (**b**) Side view of the sensor model with the capacitance of each layer.

The flow of the resin can be modelled as a thin dielectric layer which can move along the length direction (L) on the top surface of the sensor. Figure 2a illustrates the sensor geometry in perspective view and Figure 2b presents the side view of the sensor model with the resin movement shown on the top surface. Total capacitance is the sum of the three following capacitances in Equation (2) [39].

(2) $$C_{total}=C_{resin,dielectric}+C_{air,dielectric}+C_{dielectric}$$

where $C_{resin,dielectric}$ and $C_{air,dielectric}$ are calculated with Equation (1) for two dielectric materials and $C_{dielectric}$ is a constant coplanar capacitance for one dielectric layer. The function that describes the change of the capacitance with resin frontal flow is given by Equation (3).

(3) $$C_{total}=\varepsilon_{0}\varepsilon_{eff\_r}\cdot \frac{K\left(k_{0}^{\prime }\right)}{K\left(k_{0}\right)}\cdot x+\varepsilon_{0}\varepsilon_{eff\_a}\cdot \frac{K\left(k_{0}^{\prime }\right)}{K\left(k_{0}\right)}\cdot \left(L-x\right)+\varepsilon_{0}\varepsilon_{eff\_d}\cdot \frac{K\left(k_{0}^{\prime }\right)}{K\left(k_{0}\right)}\cdot L$$

where $\varepsilon_{eff\_r}$,$\;\varepsilon_{eff\_a}$ and $\varepsilon_{eff\_d}$ are the effective relative permittivity of the resin, air and dielectric materials respectively, L is the length of the sensor and x the resin flow.

Sensitivity is a measure of the sensor signal strength [28] and it is defined as the ratio of the variation of the capacitance to the initial capacitance [25]. It is dependent on the capacitance of the materials that are on the top of the sensor surface. The calculation, which is derived by Equation (4) uses the initial capacitance ($C_{0}$) between the driving and sensing electrodes and the capacitance changes (ΔC) due to different materials. In this case study, the impregnation of the carbon fibre tows by the resin contributes to the capacitance change.

(4) $$Sensitivity=\frac{\Delta C}{C_{0}}\cdot 100\%\overset{\;}{\Rightarrow }Sensitivity=\left|\frac{C_{resin}-C_{air}}{C_{air}}\right|\cdot 100\%$$

where $C_{resin}$ is the derived capacitance when the epoxy resin is on the top of the sensor and $C_{air}$ is the initial capacitance, before the resin reaches the sensor.

### 2.2. Electrostatic Numerical Modelling

Numerical modelling of the sensor enhances the understanding of the electric field response to changes in material properties (dielectric permittivity and conductivity) and sensor geometry. The electrostatic behaviour of the proposed sensor has been analysed using the Partial Differential Equations (PDE) toolbox in the MATLAB (R2019a) software, which can be used to solve the electrostatic field equations [29]. For the experimental set-up used to test the sensor, the measuring equipment that was used for the resin flow monitoring operated at low frequencies (below 10 kHz). Previous numerical studies for low frequencies indicate that the materials were in a quasi-equilibrium state [40,41]. In this state, electrodes behave in a resistive way and dielectrics in a capacitive way. Thus, the electrostatic Poisson’s equation of the elliptic type, which means that it is time-independent, satisfies a solution for the problem. Equations (5) and (6) were used to solve the electrostatic problem. A 2D analysis was used, where the materials are assumed to have homogeneous behaviour along the length of the sensor. Thus, the 2D electrostatic modelling was considered adequate enough to provide the information on the capacitance and the optimization of the sensor geometry [41].

(5) $$\nabla \cdot \vec{D}=-\rho_{v}\overset{\vec{D}=\varepsilon \cdot \vec{E}}{\Rightarrow }\nabla \cdot \left(\varepsilon \cdot \vec{E}\right)=-\rho_{v}$$

(6) $$\vec{E}=-\nabla \;V$$

where ε is the coefficient of dielectric permittivity, $\rho_{v}$ is the space charge density, V is the scalar potential, $\vec{E}$ is the vector of the electric field and $\vec{D}$ is the vector of the electric displacement field. The simulation provides the solution of the voltage distribution, the electric field and electric displacement field on a defined geometry. The capacitance was derived by the electric field energy, as described in Equations (7) and (8) [34]:

(7) $$U=\frac{1}{2}\underset{V}{∭}\vec{D}\cdot \vec{E}\cdot dV$$

(8) $$C=\frac{2\cdot U}{v^{2}}$$

where U is the electric field energy, v is the voltage difference between the two sensor electrodes. The electric field energy U is the integral of the volume V that contains the sensor geometry.

**Figure 3:**
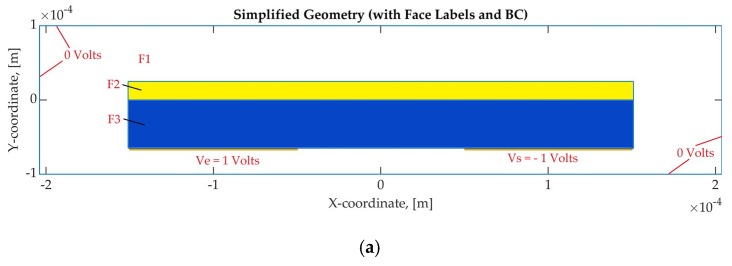

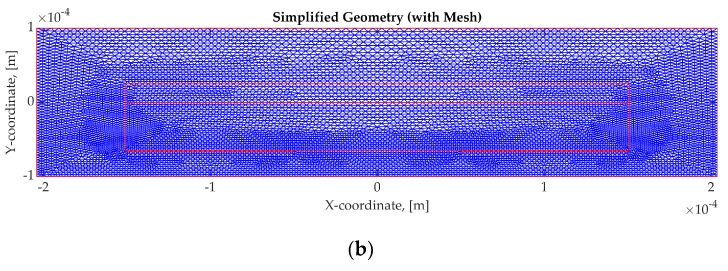
Geometry of the simplified electrostatic model; (**a**) Domains and boundary conditions applied at the edges and (**b**) Mesh.

Figure 3a shows the simplified model with the simulation space (F1), the resin-thick layer (F2) and the insulation dielectric polyimide tape (Tesa 51408 [42]) (F3) that are used for the modelling. The simplified coplanar model includes the rectangular shape electrodes and the dielectric materials on the top and of the sensor surface. The thickness of the materials is 65 μm for the tape and 25 μm for the resin layer. As previously explained, CMT analysis considers zero thickness electrodes. Thus, the electrodes thickness was set to 1 μm, which was adequately small compared to the model dimensions. Figure 3b presents the simplified geometry and the mesh with the quadratic elements that were used for this simulation. For the model presented in Figure 3, both electrode gap and electrode width are 100 μm. Boundary conditions (BC) were applied on the edges of the model, as illustrated in Figure 3a. The electric potential of the driving electrode is a known variable and was used to select Dirichlet boundary conditions. Equation (9) describes the Dirichlet boundary conditions, as they are specified by the PDE toolbox [29].

(9) h⋅u=r

where h and r are functions of the electric potential. Thus, u is the initial electric potential that was applied at the model edges. In the case of time-dependent models, parameter r can be a function of time. To simplify the model, these parameters were constants [29]. In this case study, the grounded edges of the simulation space have zero potential (u=0 Volts). The electrode ($V_{e}$ in Figure 3a) to which voltage is applied has an electric potential equal to 1 Volt (u=1 Volts). The sensing electrode ($V_{s}$ in Figure 3a) has an electric potential equal to −1 Volt (u=−1 Volts).

Dielectric properties of the materials that were used for the electrostatic model were measured with the Portable Dielectric Measurement Kit (PDMK) from Pueschner Gmbh [43]. This system includes a microwave source, which generates an excitation with a frequency sweep in the range of 1.5–2.45 GHz and calculates the dielectric permittivity and loss factor. The real part of the dielectric permittivity (ε) is the coefficient that describes the material properties in the elliptic type Poisson’s Equation (5). Table 2 includes the coefficients that were used in the simulation. The resistive part of the materials, which is related to the materials loss factor, was excluded from the current analysis due to the low-frequency measuring equipment that was used for flow monitoring.

### 2.3. Sensor Prototype

In many cases, during the initial stage of the composite manufacturing processes, the heating of the tool is required which depends on the materials used (epoxy resin and pre-form) [44]. Therefore, the use of high-temperature, robust and flexible materials is mandatory for the integration of the sensor to the process. However, this constraint limits the available options for the selection of the dielectric layers. The prototype sensors were manufactured using Rogers XT/Duriod 8000 high frequency dielectric material with double copper clad [45]. This material was selected due to its dielectric and thermal properties, and suitability for use in the composite manufacturing process.

The copper etching technique with ferric chloride CIF AR371 [46] was used to remove copper between the electrodes and create the desired electrode gap according to the design. Different sensor designs were tested with variations of the electrode gap and width. An additional layer of a high temperature polyimide tape (Kapton–Tesa 51408) was placed on both sides of the sensor to prevent the electrodes from coming into contact with any other conductive elements such as carbon fibres and the metallic tool. This configuration resulted in a final sensor thickness of 215.8±0.5 μm. The dielectric layer thickness affects the sensor’s capacitance and as there are limitations from the materials used for the sensor, it was decided to examine the addition of a resin permeable dielectric layer, or surfacing veil, that could be integrated into the infusion process. The effect of the geometric characteristics and the surfacing veil to the sensor response was performed with the capacitance calculation and measurement.

**Figure 4:**
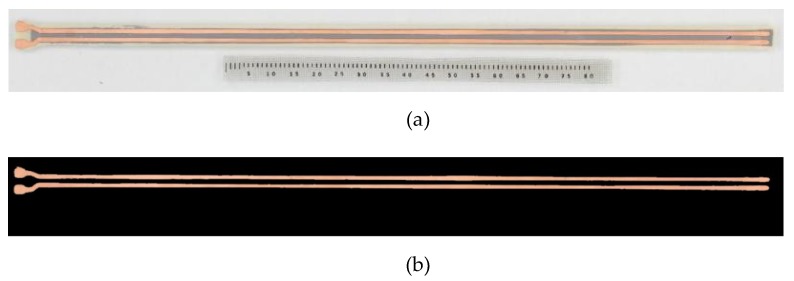
Image analysis for the geometric characteristics calculations. (**a**) Image of the sensor taken with a macrographic camera; (**b**) Masked red-green-blue (RGB) image using the L*a*b colour space thresholding.

An image-processing algorithm was developed to extract the actual geometric characteristics of each sensor that was manufactured. This was performed in order to capture the inevitable imperfections which can occur due to the copper etching technique. The accuracy of the actual geometry is important for the validation of the sensor’s electrostatic modelling and characterisation. Figure 4a illustrates the sensor geometry which was used for the image analysis and Figure 4b shows the masked RGB image obtained after the use of the L*a*b colour space thresholding [47,48].

In order to avoid potential errors in the segmentation of the electrodes with the threshold values, a sensitivity analysis was performed [49]. The initial colour-space (L, a, b) values were obtained with the Image Processing toolbox in the MATLAB (R2019a) software [50]. The developed algorithm used the initial colour space values to calculate the electrode size (s) and gap (2g). Afterwards, the colour-space values were updated with the use of a random number generator, and the same calculation is performed with the updated threshold values. The variation of the (L, a, b) values alters the segmentation of the electrodes and the calculation of the sensor’s characteristics. This can be seen in Table 3 which includes the geometric characteristics (electrode width and gap) of the manufactured dielectric sensors ($S_{i},$ i = 1–4) with the mean (M) and the standard deviation (SD) of the calculated values from the image-processing algorithm.

### 2.4. Experimental Set-Up

The validation of the numerical model and an assessment of the sensor’s performance for monitoring resin flow was evaluated using a flat composite panel vacuum infusion set-up. This set-up included four sensors to monitor the capacitance changes due to the flow of resin through a dry fabric pre-form. Consistency of the values obtained is important for the validation. The experimental set-up was used to maintain constant process conditions during vacuum infusion and examine the ability of the sensors to monitor the capacitance changes. The resin outlet was connected to a pump to maintain vacuum during the infusion phase and the temperature was maintained at ambient conditions for both the infusion and the cure phases, as specified by the resin manufacturing specifications. Different experimental conditions, for example with the use of another resin where heat is applied, could cause the sensor’s response to deviate from the one studied in this paper.

**Figure 5:**
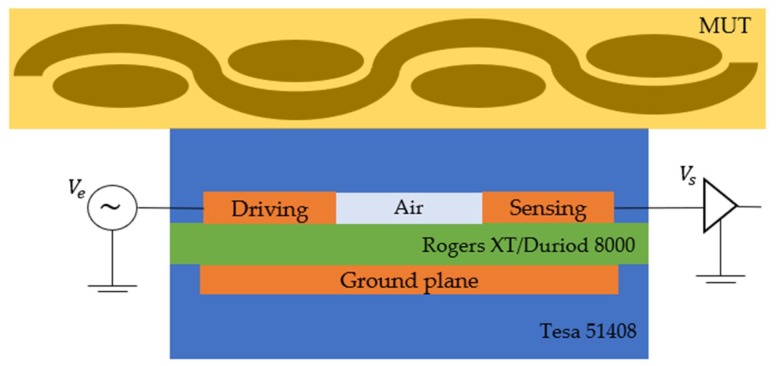
Electrode description and their connectivity to the measuring system.

The dielectric measurement system, DieMOS from EtS Ltd. was used to acquire the capacitance values of the sensors during the infusion process. DieMOS can measure four sensors simultaneously and the manufactured sensors were ordered into two groups, as shown in Table 3. The system operates with the I-V measuring method [51] to determine the impedance in the frequency bandwidth of 1 Hz to 10 kHz. An equivalent circuit with a resistance and a capacitance connected in parallel was used to calculate the real-time dielectric parameters. The excitation voltage of the sensors was set to 1 Volt. The excitation was a frequency sweep which was recorded in 12 points for the bandwidth 1 Hz to 10 kHz. Figure 5 illustrates the sensor configuration and materials, the electrodes type and their connectivity to DieMOS. The excitation was connected to the driving electrode ($V_{e}$) and the sensing electrode ($V_{s}$) with a coaxial cable to the data acquisition unit. As previously discussed, a ground plane was used to shield and reduce interference with other electric fields.

The resin flow length calculation, which corresponds to the sensor coverage, was performed with Equation (10).

(10) $$L_{measured}\left(t\right)=\frac{C\left(t\right)-C_{air}}{C_{resin}-C_{air}}\cdot L$$

The equation considers the capacitance changes, the initial capacitance ($C_{air}$), the capacitance when resin covers the sensor is ($C_{resin}$) and the total sensor length (L). The length ($L_{measured}$) is the calculated flow length that was derived by the measured capacitance changes during the infusion progress. The actual flow front, which was recorded with a digital camera, was compared with the calculated flow front using the dielectric signal to evaluate the sensor’s performance.

**Figure 6:**
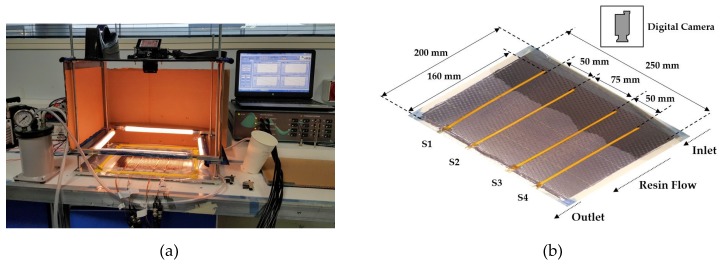
(**a**) Picture of the experimental set-up for the capacitance measurements during the infusion process; (**b**) Snapshot of the infusion process and the materials used.

The materials used in this experimental set-up were one layer of carbon fibre G0926 from Hexcel, a two-part epoxy resin IN2 with the slow hardener from Easy Composites Ltd., and a surfacing veil E-Glass V1-030 from Fiberlink [52]. Figure 6a illustrates the experimental set-up which includes a flat panel rig with the composite material, a lighting system and the dielectric system. Figure 6b presents a snapshot of the setup showing the dimensions of the carbon fibre pre-form, the four sensor locations and the resin flow direction.

## 3. Model Validation and Results

### 3.1. Initial Model Validation—Comparison of the Simplified Sensor Geometry

**Figure 7:**
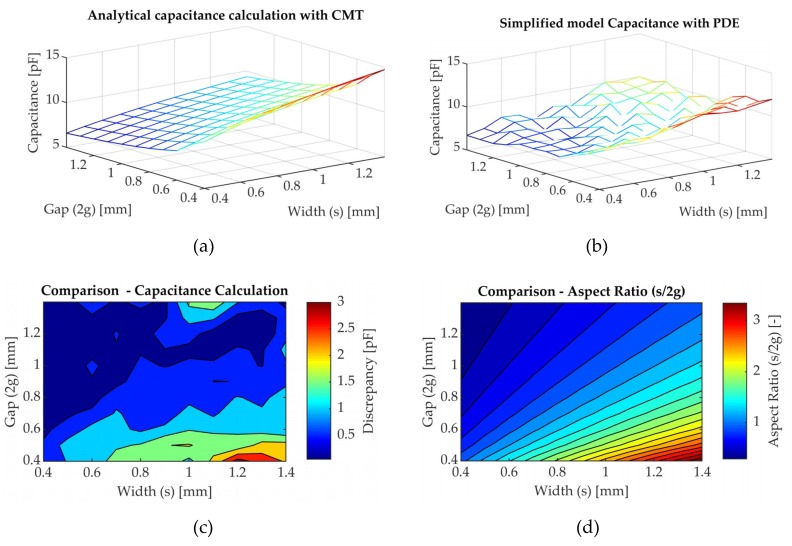
Comparison of the analytical conformal mapping technique (CMT) and Partial Differential Equations (PDE) model for the simplified sensor geometry and the relative error with regards to the geometric characteristics; (**a**) Capacitance [pF] with the analytical CMT; (**b**) Capacitance [pF] with the PDE; (**c**) Discrepancy of the capacitance [pF]; (**d**) Aspect ratio map [-].

The simplified 2D analytical model was used to compare sensor geometry with the electrostatic numerical model. Afterwards, the capacitance of the sensor was calculated. Coplanar electrodes sensor configuration with two dielectric layers was presented in Figure 1 and is examined in this section. A comparison of the calculated capacitance for the simplified geometry (Figure 1 and Figure 3) for the analytical and the electrostatic model is presented in Figure 7.

The case study used for the comparison includes the parameters of Table 1 and the dielectric permittivity values that were previously presented in Table 2. The range of values for the electrode gap (2g) and width (s) were defined by the sensor manufacturing limitations and its integration to the process. The minimization of the degree of intrusiveness of the sensor to the process and the final part quality is one of the attributes to be considered for the proposed sensor as employed in a liquid infusion process. Thus, the upper limit for both sensors’ geometric characteristics was set to 1.4 mm. Figure 7a,b show the capacitance that were calculated with the CMT analytical method for two dielectric materials and with the PDE electrostatic numerical modelling. Figure 7c illustrates the discrepancy in the capacitance derived from this comparison and Figure 7d a map of the geometry aspect ratio (s2g).

An important aspect to be considered for the sensor design is the signal-to-noise ratio (SNR). For the dielectric sensors, the capacitance is a dominant feature to sensor’s SNR which can influence its robustness [26]. The geometric characteristics clearly affect the capacitance, which can be observed for both the CMT and PDE solutions. The capacitance drops when the gap between the electrodes increases and an increase in the electrode width results in a larger capacitance. This behaviour is to be expected and it has been explained by Nassr et al. [34] and Igreja et al. [30]. The capacitance calculation in the PDE model is sensitive to the mesh and this can be seen in Figure 7b with the non-smooth results. As presented by Bhuiyan et al. [53], the comparison of the analytical and numerical calculation for the coplanar electrodes geometries is related to the geometry aspect ratio. The capacitance difference, as shown in Figure 7c, appears to become significant for the geometries with aspect ratio higher than two (Figure 7d). The discrepancy of the capacitance calculation between the CMT and PDE decreases when the electrodes width (s) is below 0.8 mm and increases for geometries with electrodes gap (2g) below 0.7 mm and electrode width higher than 0.8 mm. The dependency of the geometric characteristics aspect ratio to the numerical calculation with the PDE is highlighted in Figure 7c,d.

### 3.2. Sensor Electrostatic Model and Capacitance

The calculation of the capacitance for complex sensor geometries, such as the one presented in this publication is usually performed with numerical models. The detailed sensor model includes the simplified geometry with the addition of the ground electrode. The worst case in terms of the resin-rich areas is when the carbon fibre tows are perpendicular to the sensor’s length. Thus, the carbon fibre tows are simulated as a thin conductive layer. The dielectric thickness of the Kapton tape is 65 μm and the resin-rich layer is approximately the size of a carbon fibre tow diameter which were both set to 25 μm [54]. The modelled sensor geometry has the same geometric characteristics as the manufactured sensors, with the electrode thickness set to 17.5 μm and the dielectric layer between the electrodes and the ground plane set to 50.8 μm, as specified by the manufacturer [45].

As previously reported in Section 2.4, the surfacing veil E-Glass V1-030, was used as an additional layer between the sensor and the carbon fibre. The use of this material depends on the manufacturing process and its impact on the final composite part. In this case study, the resin impregnates the surfacing veil layer and the resin layer is increased to a thickness of 100 μm.

**Figure 8:**
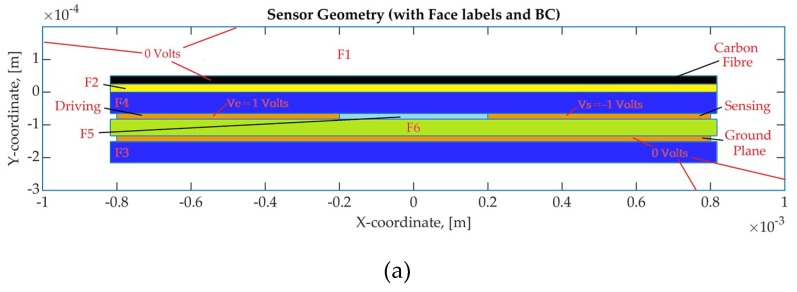

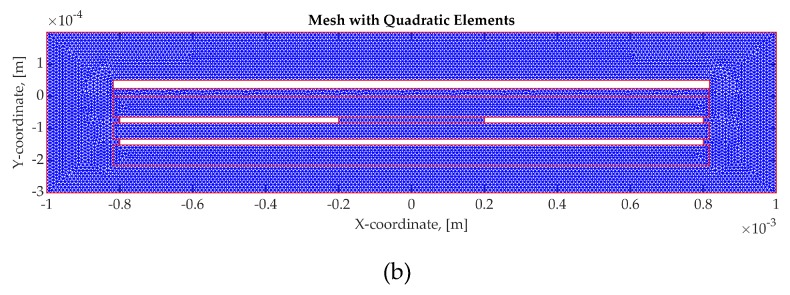
Sensor geometry (**a**) Domain labels; (**b**) Mesh with quadratic elements.

An overview of the domains of the numerical model and their respective properties are included in Table 4. Figure 8a illustrates the sensor geometry with the face labels and boundary conditions that were used for the electrostatic numerical simulation and Figure 8b the mesh with the quadratic elements. The geometry shown in Figure 8 includes the ground plane, the dielectric materials which were used for the prototype sensor (Table 4), the resin-rich layer and the carbon fibre layer. The modelled geometry has an electrode gap of 40 μm and a width of 60 μm.

**Figure 9:**
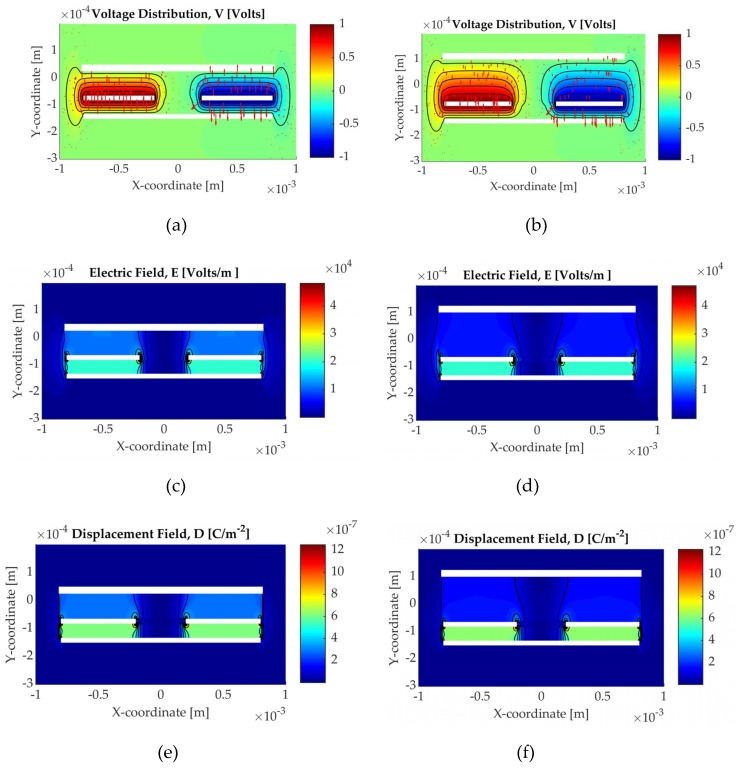
Electrostatic simulation results for the two sensors case studies with the IN2 epoxy resin; (**a**) Resin-rich layer; (**b**) Veil–voltage distribution [Volts] with the electric field vectors; (**c**) Resin-rich layer; (**d**) Veil–electric field magnitude [V/m]; (**e**) Resin-rich layer; (**f**) Veil–electric displacement field magnitude [C/m^−2^].

The simulation results with the epoxy resin as the medium between the sensor and the carbon fibre material are presented in Figure 9 for the two cases studies. The two case studies involve the resin-rich layer and the addition of the surfacing veil. Figure 9a,c,e present the sensor geometry with the resin-rich layer case and Figure 9b,d,f with the surfacing veil. The voltage distribution with the electric field vectors (Figure 9a,b), the amplitude of the electric field (Figure 9c,d) and the electric displacement field (Figure 9e,f) are illustrated.

The voltage distribution and the electric field are related to the sensor response and the change in material. The amplitude of both the electric field and the electric displacement field are used for the calculation of the energy and, afterwards, the calculation of the capacitance, as described in Equations (7) and (8). As can be observed in Figure 9, the addition of the glass fabric alters the electric field and consequently this has an effect on the capacitance.

**Figure 10:**
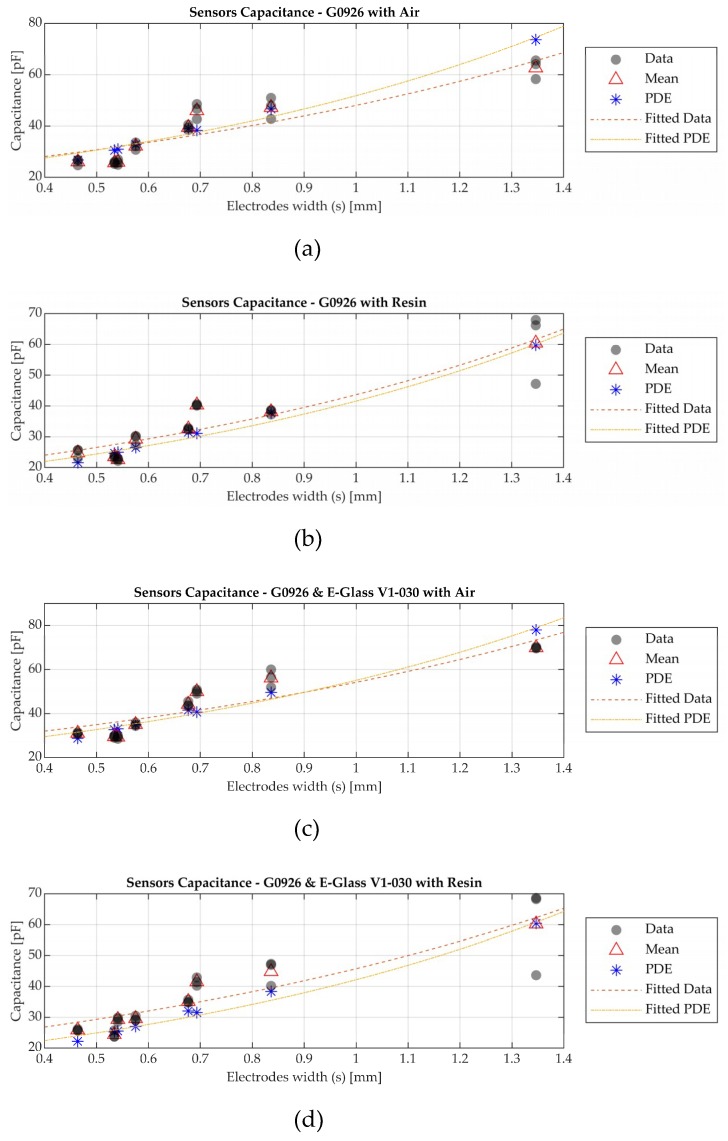
Sensors capacitance of the experimental data and PDE model values. (**a**) Dielectric sensor–air; (**b**) Dielectric sensor–epoxy resin; (**c**) Dielectric sensor with surfacing veil–air; (**d**) Dielectric sensor with surfacing veil–epoxy resin.

**Figure 11:**
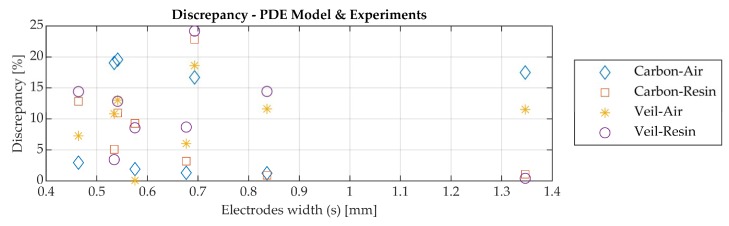
Discrepancy percentage of the experimental and PDE model values for the prototype sensors.

The effect is highlighted in Figure 10, where the capacitance of the manufactured sensors (Table 3) arranged in ascending electrode width (s) is presented. The experimental data with the average value and capacitance which was calculated with the PDE model are presented. Figure 10a presents the capacitance for the dielectric sensors when air is on the top of the sensor surface and Figure 10b is the result for resin on the top surface. Accordingly, Figure 10c and d represent a similar analysis for the dielectric sensor with the surfacing veil. Figure 11 illustrates the discrepancy percentage of the average experimental values and those calculated with the PDE simulation model for the previously mentioned case studies.

Despite the discrepancies between the modelled and measured values, presented for both air and resin cases in Figure 11, the capacitance in all cases shows a similar trend between the PDE model and experimental data (Figure 10). The discrepancy percentage is below 25% in all cases for the sensors. In addition, it can be observed that the permeable glass fabric, which can be considered as an extra dielectric layer, leads to an increase of the sensor’s capacitance. However, there is a limitation to the electrode thickness and size due to the waste of electrical energy [28]. As previously mentioned in Section 2.1, capacitance is one of the features to be considered for the sensor design.

### 3.3. Optimization Based on Sensitivity

**Figure 12:**
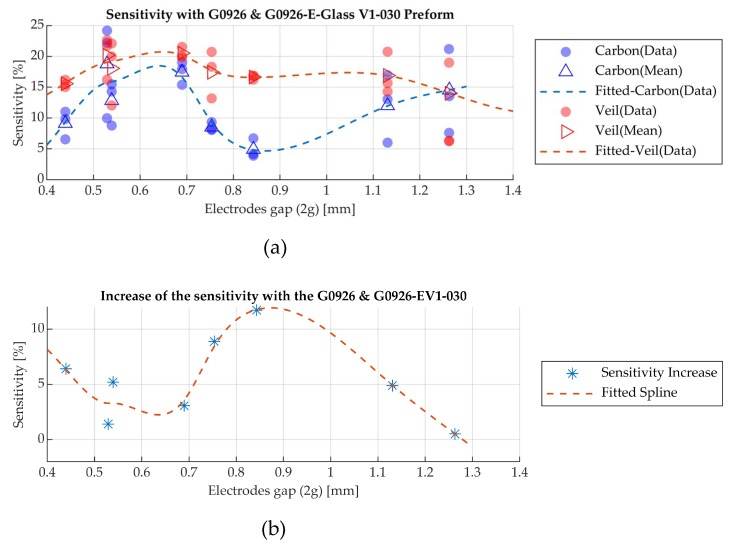
(**a**) Sensitivity based on the experimental results with the carbon fibre pre-form (G0926) and the surfacing veil (EV1-030); (**b**) Actual increase of the sensitivity with the additional surfacing veil.

Electrostatic modelling of the sensor and the experimental values were used to calculate the capacitances in each case study, as previously presented, and Equation (4) was used to calculate the sensitivity. Figure 12a illustrates the sensitivity values of the manufactured sensors measured with the experimental set-up for the two cases studies and Figure 12b is the actual increase of the sensitivity with the addition of the surfacing veil.

As presented in Table 3, the prototype sensors have different geometric attributes and in Figure 12a the sensitivity values are presented in ascending electrode gap (2g) which highlights that the geometry has an effect on this characteristic. The highest average in the sensitivity for the carbon fibre pre-form was found to be at 18.7% and was obtained by sensor S2 in Group B (Table 3). Accordingly, sensor S3 in Group B (Table 3) recorded 20.5% for the glass-carbon pre-form. Also, the experimental results and Figure 12b indicate that the addition of the surfacing veil leads to an increase of the sensitivity in the range of 1% to 12% and this is also affected by the sensor’s geometric characteristics. Sensor S1 in Group A (Table 3) was affected the most with the addition of the glass surfacing veil layer, as the actual increase in the sensitivity was 11.7%.

### 3.4. Sensor’s Evaluation Based on Visual Data

**Figure 13:**
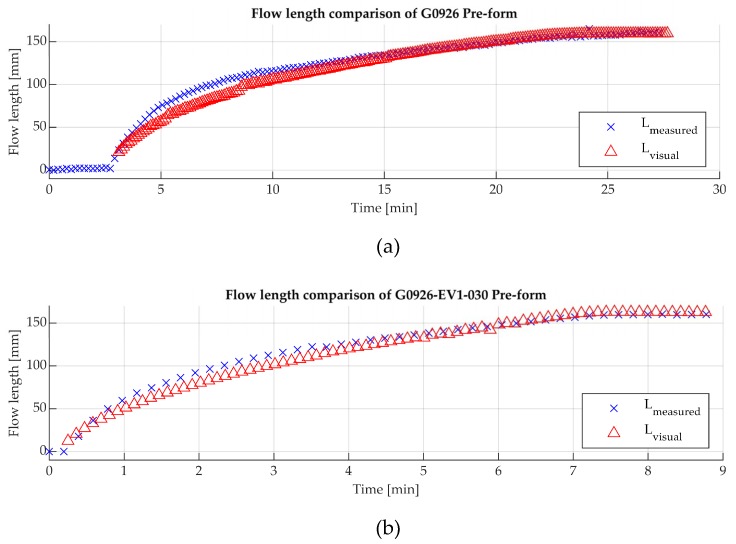
Comparison of the measured ($L_{measured}$) and actual ($L_{visual}$ ) resin flow during the vacuum infusion process. (**a**) Experiment with carbon fibre pre-form; (**b**) Glass-carbon fibre pre-form.

The sensors performance for measuring the flow front was evaluated by comparing with the visual flow using Equation (10). Figure 13 presents an example of the flow length comparison with the measured and actual flow. Figure 13a,b illustrate the carbon fibre and glass-carbon fibre case studies respectively.

As can be seen in Figure 13, the estimated flow length provides good agreement with the actual flow. However, in order to evaluate the sensor’s performance, the discrepancy of this estimation was calculated using the root mean square error (RMSE). This analysis was performed for the conducted experiments and sensors. Table 5 presents the discrepancy percentage of the RMSE between estimation and actual flow length for the two examined case studies. Table 5 includes mean (M) and standard deviation (SD) of the derived RMSE values.

The maximum average discrepancy percentage was 16.4% for the sensor S3 in Group A, which translates to 2.6 cm difference of the measured and actual flow. Sensors S2 and S3 in Group B, which recorded the highest sensitivity values, had the best performance in the resin flow estimation for both pre-forms, as presented in Table 5. The average discrepancy in the flow length during the infusion of the carbon fibre pre-form was 0.25 cm and 0.46 cm respectively for these sensors.

## 4. Discussion

The analytical model using CMT, which includes the main geometric characteristics of the sensor capacitance and the resin flow, was introduced in Equations (1)–(3). The analytical model includes two dielectric materials and the coplanar electrodes. This geometry is an approximation of the actual sensor, as the prototype sensor includes an additional ground electrode. An electrostatic model with the use of PDE which includes the dielectric properties of the materials used was developed. The analytical model and a simplified electrostatic model are compared with regard to the capacitance calculation. The comparison results indicate that there is a discrepancy of the calculation in the geometries with aspect ratio higher than twice that of the electrodes’ distance and width (Figure 7c,d). The use of an electromagnetic software and the 3D model of the sensor is suggested to minimize discrepancies in the capacitance calculation. Temperature effect and the lossy character of the dielectrics used should be investigated and integrated into the model. This can provide an estimation of the sensor’s response with the use of different materials to the ones used in this study.

The geometric attributes of the manufactured sensors were obtained with an image-processing algorithm. Their performance to monitor the resin frontal flow was assessed with an experimental set-up which was used to acquire the capacitance values during the infusion process. The methodology of sensing the flow with dielectric sensors becomes challenging with the use of carbon fibre materials due to their conductivity. Therefore, it is important to maximize the sensitivity of the sensor with regard to the materials used by carefully considering the design of the sensor, which will lead to the development of a reliable flow-monitoring system. The methodology that was used for the resin flow monitoring considers the dielectric signal and the changes during the infusion process. Equation (10) estimates resin’s flow length which is related to sensor’s coverage with the liquid resin. The validation of this estimation and the sensor’s performance were assessed with the comparison of the measured and visually obtained flow and calculation of their discrepancy with the RMSE values.

The simulated and measured capacitance values of the manufactured dielectric sensors are compared in Figure 10. The discrepancy percentage varies with the sensor design and the investigated materials. However, as presented in Figure 11, the discrepancy percentage was below 25% for all case studies. As previously mentioned, using a numerical model which includes frequency and temperature effect of the properties of the materials can reduce the percentage in the discrepancy.

The experimental trials were used to calculate the sensors’ sensitivity for the two presented pre-forms. The sensitivity map of the prototype sensors indicates that alterations of the geometry lead to different sensitivities for the same pre-form. Also, the addition of the surfacing glass fabric veil increased the sensitivity in all sensor geometries. The sensitivity was increased up to 12% with the addition of the surfacing glass fabric.

## 5. Conclusions

A linear flexible dielectric sensor was designed to monitor the resin flow during the vacuum infusion process with a carbon fabric pre-form and a multi-layer carbon-glass fabric pre-form. The sensor was placed at the surface of the pre-forms. The prototype sensors were manufactured with the flexible dielectric material Rogers XT/Duriod 8000 which was covered by the polyimide tape Tesa 51408. Their performance was evaluated with an experimental set-up and the results demonstrate their applicability to measure the resin flow with carbon fibre materials. The evaluation revealed that the measurement accuracy varies from 1.6% to 16%, which is affected by the sensor’s design. The integration of the sensor to a pilot RTM line is the next stage of this research and is part of a future publication.

These sensors provide an advantage compared to other sensor technologies as they can be used with complex shape geometry moulds and minimize their intrusiveness in the process and the quality of the final composite part. The results presented in this study indicate that the design of the sensor’s geometric characteristics is important for the robustness of its response and integration to the infusion process. Thus, the sensors’ modelling and investigation of the selected dielectric materials can overcome the limitations of their usage to conductive pre-forms and increase their sensitivity.

## Figures and Tables

**Table 1:** Geometric and material parameters used for the sensor analysis.

Parameter	Description	Units
2g	Distance of the electrodes	mm
s	Electrodes width	mm
$h_{1}$	Thickness of dielectric layer	mm
$h_{2}$	Thickness of resin layer	mm
$\varepsilon_{1}$	Dielectric permittivity of dielectric layer	-
$\varepsilon_{2}$	Dielectric permittivity of resin-rich layer	-

**Table 2:** Dielectric coefficients of the materials used for the simplified electrostatic study.

Material Name	Dielectric Permittivity	Domain
Air	1.00059	F1
Epoxy Resin (IN2)	2.832	F2
Polyimide tape (Tesa 51408) [42]	3.12	F3

**Table 3:** Geometric characteristics of the manufactured dielectric sensors (dimensions [mm]).

Characteristic	Width (s)		Gap (2g)		Width (s)		Gap (2g)	
Sensor	Group A				Group B			
	M	SD	M	SD	M	SD	M	SD
S1	0.47	0.11	0.84	0.11	1.35	0.09	1.25	0.10
S2	0.53	0.04	0.75	0.03	0.57	0.10	0.52	0.10
S3	0.54	0.08	0.54	0.09	0.69	0.06	0.69	0.07
S4	0.67	0.04	0.44	0.06	0.83	0.08	1.13	0.07

**Table 4:** Dielectric coefficients of the materials used for the sensor’s electrostatic study.

Material Name	Dielectric Permittivity	Domain
Air	1.00059	F1, F5
IN2 Epoxy Resin	2.832	F2
Tesa 51408 Polyimide tape [42]	3.12	F3, F4
Rogers XT/Duriod 8000 [45]	3.54	F6

**Table 5:** Flow discrepancy percentage [%] analytics for the two case studies and prototype sensors.

Pre-form	G0926		G0926-EV1-030		G0926		G0926-EV1-030	
Sensor	Group A				Group B			
	M	SD	M	SD	M	SD	M	SD
S1	8.2	2.6	7.7	1.7	9.3	5.1	16.1	4.0
S2	7.8	3.0	3.8	0.5	1.6	0.4	2.2	0.8
S3	16.4	3.4	10.1	5.3	2.9	0.5	3.4	0.6
S4	5.2	1.4	6.0	1.5	6.7	1.2	11.1	1.0
